# Iron reference intake values for 7- to 23-month-old Brazilian children

**DOI:** 10.1017/S1368980025101018

**Published:** 2025-10-28

**Authors:** Alessandra da Silva Pereira, Inês Rugani Ribeiro de Castro, Eliseu Verly-Junior

**Affiliations:** 1 Escola de Nutrição, Universidade Federal do Estado do Rio de Janeiro, Rio de Janeiro, Brasil; 2 Instituto de Nutrição, Universidade do Estado do Rio de Janeiro, Rio de Janeiro, Brasil; 3 Instituto de Medicina Social, Universidade do Estado do Rio de Janeiro, Rio de Janeiro, Brasil

**Keywords:** Nutrient reference values, Iron, Bioavailability, Children

## Abstract

**Objective:**

To adapt current iron intake reference values for Brazilian children aged 7–11 and 12–23 months, using the opportunity to apply the principles and rationale of the harmonisation approach.

**Design:**

Nutrient reference values (NRV), including the average requirement (AR) and population reference intake (PRI), were estimated for children aged 7–11 and 12–23 months. We applied and adapted methods from the Institute of Medicine (IOM) and the European Food Safety Authority (EFSA) to estimate the NRV. Body iron losses, iron needs for growth and dietary iron bioavailability were estimated using both local and external data.

**Setting:**

Rio de Janeiro, Brazil.

**Participants:**

Data on dietary intake from a probabilistic sample of children aged 7–23 months in the city of Rio de Janeiro, Brazil, were used to estimate iron consumption and bioavailability.

**Results:**

The mean physiological iron requirements were 0·78 mg/d (7–11 months, male), 0·53 mg/d (7–11 months, female), 0·79 mg/d (12–23 months, male) and 0·54 mg/d (12–23 months, female). Mean dietary iron bioavailability was 7·5 % across all age and sex groups. AR and PRI were 10 mg/d and 19 mg/d for children aged 7–11 months, and 7 mg/d and 13 mg/d for those aged 12–23 months. NRV did not differ by sex.

**Conclusion:**

The AR for children aged 7–11 and 12–23 months were 11 mg/d and 8 mg/d, respectively. The corresponding PRI were 20 mg/d and 14 mg/d. The estimated Brazilian NRV were higher than those of the IOM and EFSA. Iron bioavailability was the most influential factor explaining the differences from other NRV.

Nutrient reference values (NRV) for a population are essential for various applications in clinical nutrition, public health and policy development^(1,2)^. In Brazil, for example, several initiatives are based on NRV. The School Meals Food Program provides funds to public schools, where menus must meet specific percentages of the reference values for key nutrients^(3)^. The mandatory fortification of wheat and corn flour with iron and folic acid to prevent anaemia and neural tube defects was also based on population reference values^(4)^. Additionally, NRV play a crucial role in supporting legal regulations on nutrient content, health claims and complementary labelling, such as Normative Instruction No. 76, published by the Brazilian National Health Surveillance Agency^(5)^.

Most NRV have been established in high-income countries, with few exceptions such as Venezuela^(6,7)^ and India^(8)^. Consequently, nutritionists, clinicians, researchers and policymakers in low- and middle-income countries often rely on external NRV, such as the dietary reference intakes (DRI) from the US Institute of Medicine (IOM)^(9)^. The DRI are a set of reference values developed for the USA and Canada, which include the estimated average requirement (EAR), RDA, adequate intake (AI) and tolerable upper intake level (UL)^(10)^. The development of the DRI and other NRV is based on available scientific evidence, selected health and metabolic indicators of nutrient adequacy, and – where applicable – the estimated bioavailability of nutrients from typical dietary patterns^(10)^. Another expert body involved in developing NRV is the European Food Safety Authority (EFSA)^(2)^. Although EFSA shares several methodological assumptions with the DRI framework, it also considers specific characteristics of the European population.

Physiological nutrient requirements vary slightly across populations, and it is feasible to adapt recommendations from different sources to harmonise core reference values^(11)^. However, local characteristics can significantly influence nutrient intake recommendations. Therefore, it is important to establish or adapt country-specific NRV that consider social (e.g. lifestyle), demographic, anthropometric and environmental (e.g. climate) factors, as these can affect both nutritional status and consequent nutritional needs^(8)^. Within this context, iron is a nutrient of major global public health concern, with anaemia being the most common consequence of iron deficiency^(12,13)^. Iron is essential for numerous physiological functions, including oxygen transport, electron transfer, enzymatic activity and energy metabolism^(14)^.

NRV are generally established using balance studies or factorial methods. Due to a lack of data linking iron intake directly to biomarkers of physiological need, the factorial approach is commonly used to establish iron NRV^(15)^. This method estimates NRV based on body iron losses, iron requirements for growth and dietary iron bioavailability^(16)^. Body weight is a critical input in determining basal iron losses and, consequently, physiological iron requirements. In 2006, the WHO published growth standards for children and adolescents using data from both low- and high-income countries, and these have become the international standard. However, current IOM NRV were developed using the US Centers for Disease Control and Prevention (CDC) 2000 growth charts, which were based on children with a low prevalence of breast-feeding, making them less applicable to populations with different nutritional and breast-feeding patterns^(17)^.

Dietary iron exists in two forms: heme and non-heme. Heme iron has higher bioavailability (approximately 15–35 %)^(18)^ but typically accounts for only 10–15 % of total dietary iron intake. Non-heme iron, which comprises about 85–95 % of dietary iron, has lower and more variable bioavailability, ranging from 1 % to 10 %^(18,19)^. The IOM assumes a bioavailability of 10 % for children aged 7–11 months and 11 % for those aged 1–3 years, whereas EFSA assumes 10 % bioavailability across all child age groups^(20,21)^. It is important to note that animal-source foods, which are rich in heme iron, are consumed more frequently in high-income countries than in middle- and low-income countries – largely due to economic constraints^(22)^.

In 2018, following an initiative launched by the FAO and the United Nations University in 2007, a panel of experts released the Global Harmonization of Methodological Approaches to Nutrient Intake Recommendations^(23)^. Harmonising the methodologies used to derive NRV is important for several reasons: (i) it ensures the objectivity and transparency of NRV across different contexts; (ii) it provides a shared basis for reviewing nutrient needs; (iii) it facilitates access to resources by low- and middle-income countries and (iv) it supports the development of coherent nutrition and health policies^(23,24)^.

Considering the importance of country-specific NRV, this study aimed to adapt the current IOM and EFSA reference values for iron – specifically the average requirement (AR) and population reference intake (PRI) – for use in Brazilian children. This adaptation was guided by the principles and rationale of the harmonisation approach^(23)^.

## Methods

**Figure 1.**
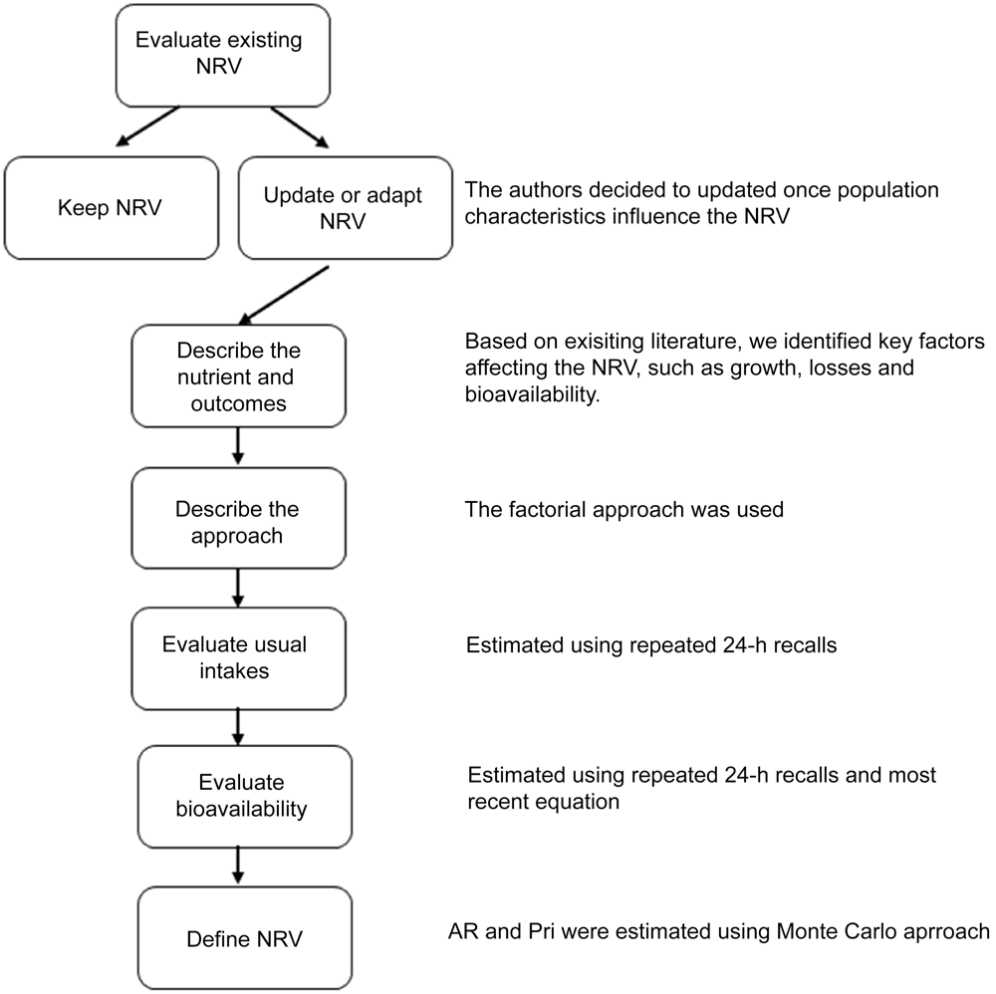
Fluxogram for adapting iron NRV for Brazilian children. NRV, nutrient reference values; AR, average requirement; PRI, population reference intake.

The procedures used to establish NRV for Brazilian children aged 7–23 months followed the approach recommended by NASEM (2018)^(23,24)^, which emphasises the harmonisation and transparency of rationales and methodological procedures. The terminologies used by NASEM for NRV include AR, PRI, AI and UL. Briefly, the key components of the harmonisation process include evaluating existing NRV and the scientific literature related to the nutrient of interest, deciding whether to establish a new NRV or adapt an existing one, describing the selected outcomes and methodological approach, and assessing usual nutrient intakes in the population. Inputs for establishing or adapting country-specific NRV should include, when available, local data from physiological, anthropometric and dietary studies. Figure 1 presents the steps involved in the adaptation of reference values.

Since the objective was to adapt an existing reference value, we considered the literature reviews conducted by EFSA and IOM and updated them with evidence published between 2001 and 2020^(20,21)^. Additionally, we searched for studies on iron metabolic parameters in the Brazilian population; however, no studies applicable to the purpose of this work were identified. We also defined the age groups as 7–11 months (≥ 7 and < 12 months) and 12–23 months (≥ 12 and < 24 months).

### Physiological iron requirement

The physiological requirement refers to the amount of iron that must be absorbed to maintain normal iron status and physiological functions. We used the factorial method^(20,21)^ to estimate the physiological iron requirement. This method involves estimating the amount of iron needed to replace losses through feces, urine and skin, as well as the additional amount required for growth^(25)^. In this approach, proposed by the IOM (2001)^(20)^ and EFSA (2015)^(21)^, the selected outcomes for defining physiological iron requirements were growth and nutrient balance (i.e. intake sufficient to compensate for basal losses).

### Estimated absorbed iron for growth

For children aged 7–11 months, we assumed 0·6 mg/d of absorbed iron was required for growth. This value represents the sum of 0·5 mg/d for increased Hb mass and 0·1 mg/d for the development of muscle and other tissues, as described by Domellöf & Hernell^(26)^ and also adopted by EFSA (2015). For children aged 12–23 months, the iron requirement for growth was estimated at 0·25 mg/d^(21)^.

### Estimated basal iron losses

Basal iron losses include losses through feces, urine, sweat and the exfoliation of skin^(27)^. We applied a previously estimated rate of 0·022 mg of iron per kilogram of body weight to calculate daily basal losses, a value also adopted by EFSA. This rate was derived from a longitudinal study using ^58^Fe as a tracer in thirty-five normal-weight infants (from Iowa, USA) aged 4–168 d and followed until 26 months of age^(27)^. The population distribution of basal losses was estimated by multiplying this loss rate by the age- and sex-specific reference body weight distribution. The LMS (Lambda, Mu and Sigma) parameters provided by the WHO growth standards were used to construct the reference weight distribution. Each percentile of the reference weight distribution was calculated using the following equation^(28)^:

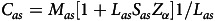




where 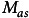 is the median weight for the age and sex *s*, *L* is lambda for the box-cox transformation, *S* is the sigma (the CV by age), 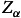 is the equivalent normal deviation for the area *α* and *C* is the centile of the reference weight to be estimated.

Based on the created weight reference distribution, the distribution of physiological iron requirement was then obtained using the equation:

### Dietary iron bioavailability

#### Dietary intake data

Iron bioavailability was estimated using dietary data (secondary data) from a cross-sectional study conducted on a probabilistic sample of 536 children aged 6–59 months, users of the Brazilian public health system (Sistema Único de Saúde – SUS), in the municipality of Rio de Janeiro^(29)^. These children were enrolled in the 2014 survey Anemia and Vitamin A Deficiency in Preschool Children: Prevalence in a Major Urban Centre and Validation of Diagnostic Methods (the VITANEMIA study). In the present analysis, data from two non-consecutive multiple-pass 24-h dietary recalls were evaluated for 261 children under 24 months of age. Most children were reported to have brown (47 %) or white (33 %) skin colour; 60 % were classified as food-secure, and the majority had a normal BMI-for-age. Further details about the original study are available in previous publications^(30–33)^. To assist parents in estimating food quantities, a booklet with household measures and food replicas was used. A national food composition database was used to quantify intake of animal and plant proteins (g), iron (mg), Ca (mg) and vitamin C (mg). Additionally, dietary phytate intake was estimated using the FAO/INFOODS/IZiNGG Global Food Composition Database^(34)^. The VITANEMIA study protocol was approved by the Municipal Health Secretariat of Rio de Janeiro (SMSRio No. 93/13).

Heme and non-heme iron contents were estimated based on the following assumptions: (i) non-heme iron corresponds to 100 % of the total iron content in non-animal-based foods (e.g. fruits, vegetables, cereals and legumes); (ii) non-heme iron corresponds to 60 % of the total iron content in animal-based foods^(35)^; and (iii) for mixed dishes (e.g. lasagna, chicken pie and sandwiches), animal-based protein accounts for 60 % of the total protein content^(36)^. A serum ferritin reference value of 25 μg/l was assumed, based on data from the National Health and Nutrition Examination Survey (NHANES) 2001–2002.

### Determination of iron bioavailability

Iron bioavailability is defined as the proportion of dietary iron that is absorbed and utilised for physiological functions. While it is generally accepted that heme iron is absorbed at a rate of approximately 25 %, non-heme iron absorption varies significantly due to the influence of numerous dietary factors^(37–41)^. No specific equation for estimating iron bioavailability in children was found in the literature. Therefore, we used the equation proposed by Armah *et al.* (2013)^(42)^, which is based on data from fifty-three adults across four studies designed to measure non-heme iron absorption from a 5-d complete diet, using an extrinsic radio iron labelling technique:

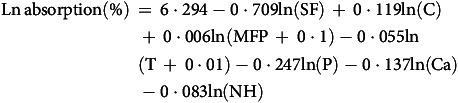




where Ln absorption is the Natural Logarithm of the absorption, SF is the serum ferritin (μg/L), C is the ascorbic acid (mg), MFP is the meat, fish and poultry (g), T is the tea (number of cups), P is the phytate (mg), Ca is the calcium (mg) and NH the is non-heme iron (mg). The tea factor was omitted because tea is not commonly reported by this age group. The total bioavailability of dietary iron was obtained by the equation:

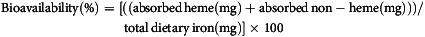




The absorption was obtained for each person per day; thus, it is subject to day-to-day variation in dietary intake. We then estimated the usual distribution of iron absorption by performing the NCI method^(43)^ using SAS codes available on the National Cancer Institute website.

### Determination of iron reference values

The distribution of dietary iron requirements was obtained by dividing the distribution of physiological iron requirements by the distribution of bioavailability. Since these distributions are uncorrelated, we performed a Monte Carlo simulation to estimate the AR and the PRI (defined as the 97·5th percentile). The simulation parameters included the mean and standard deviation of both physiological iron requirements and iron bioavailability. A synthetic population of 100 000 individuals was generated, and 10 000 Monte Carlo iterations were conducted. The resulting distributions were estimated by the previously defined age-sex groups.

## Results

Table 1 presents the mean (sd) basal iron losses and physiological iron requirements estimated for Brazilian children. Values were quite similar between sexes for both iron losses and physiological requirements. However, as expected, older children had higher estimated iron losses (0·28 mg/d for males and 0·29 mg/d for females) compared with younger children (0·18 mg/d for males and 0·19 mg/d for females).

**Table 1.** Basal losses and iron requirement for Brazilian children, by age and sex

	Males		Females	
	7–11 months	12–23 months	7–11 months	12–23 months
Iron basal losses (mg/d)				
Mean	0·18	0·28	0·19	0·29
sd	0·02	0·05	0·02	0·05
CV%	13	20	11	18
P2·5–P97·5	0·14–0·23	0·18–0·40	0·16–0·24	0·20–0·40
Physiological iron requirement^*^ (mg/d)				
Mean	0·78	0·53	0·79	0·54
sd	0·02	0·05	0·02	0·05
CV%	3	10	3	10
P2·5–P97·5	0·74–0·83	0·43–0·65	0·76–0·84	0·45–0·65

* Physiological iron requirement was obtained by summing iron basal losses (mg/d) + iron need for growth (mg/d).

Energy and nutrient intakes are shown in Table 2 for the full sample, stratified by sex and age group. Animal protein accounted for 64 % of total protein intake, and heme iron comprised 22 % of total iron intake. Although some variation was observed across sex and age groups, no statistically significant differences were detected. Similarly, heme and non-heme iron absorption varied across subgroups, but without significant differences. Total iron absorption ranged from 0·81 mg in females to 1·02 mg in males, mainly due to a higher intake of heme iron among males (3·09 mg *v*. 2·44 mg in females). Despite this, total iron bioavailability remained relatively stable across groups, ranging from 7·2 % to 8·2 %, with no significant differences. Given the minimal impact of these variations on estimated dietary requirements, and the lack of evidence for subgroup-specific differences in bioavailability, we assumed a uniform iron bioavailability of 7·5 % for the entire sample (Table 3).

**Table 2.** Energy and nutrient intakes in 7–23 months Brazilian children (*n* 261)

	Total		Female		Male		7–11 months		12–23 months	
	Mean	sd	Mean	sd	Mean	sd	Mean	sd	Mean	sd
Energy (kcal)	918	478·3	882·0	446·7	947·6	501·3	681·4	378·9	999·1	482·5
Protein – total (g)	36·0	19·6	33·7	18·3	37·7	20·4	26·7	15·7	39·1	19·8
Animal protein (g)	23·6	15·6	21·6	14·3	25·1	16·5	19·1	13·7	25·1	16·0
Vegetable protein (g)	12	8·4	12·1	8·3	12·5	8·6	7·1	6·2	13·9	8·5
Total iron (mg)	12·3	10·9	10·9	8·1	13·4	12·5	10·2	9·5	13·0	11·2
Heme iron (mg)	2·8	3·0	2·4	2·5	3·1	3·4	2·6	3·2	2·8	3·0
Non-heme iron (mg)	9·5	8·5	8·4	6·3	10·3	9·9	7·6	6·9	10·1	8·9
Vitamin C (mg)	94·4	108·2	86·4	75·0	100·7	128·4	82·9	77·0	98·3	116·8
Ca (mg)	589·1	416·5	542·3	397·9	626·0	428·3	484·8	352·7	624·4	431·1
Phytate (mg)	94·7	111·8	100·7	117·3	89·9	107·6	60·5	68·1	106·2	121·1

**Table 3.** Iron absorption and bioavailability, stratified by sex and age groups

	Male		Female		7–11 months		12–23 months		Total	
	Mean	sd	Mean	sd	Mean	sd	Mean	sd	Mean	sd
Heme Fe abs. (mg)	0·77	3·40	0·61	0·64	0·65	0·82	0·71	0·75	0·70	0·77
Non-heme Fe abs. (mg)	0·24·	0·25	0·19·	0·15	0·18	0·17	0·23	0·23	0·22	0·21
	*n*	%	*n*	%	*n*	%	*n*	%	*n*	%
Non-heme bioavailability (%)	2·4	0·5	2·35	0·43	2·55	0·48	2·31	0·42	2·37	0·45
	Mean	sd	Mean	sd	Mean	sd	Mean	sd	Mean	sd
Total Fe abs. (mg)	1·02	1·04	0·81	0·74	0·84	0·95	0·95	0·91	0·92	0·92
	%	*n*	*n*	%	*n*	%	*n*	%	*n*	%
Total bioavailability (%)	7·7 %	3·5	7·2	3·3	8·2	4·1	7·2	3·1	7·5	3·4

**Figure 2.**
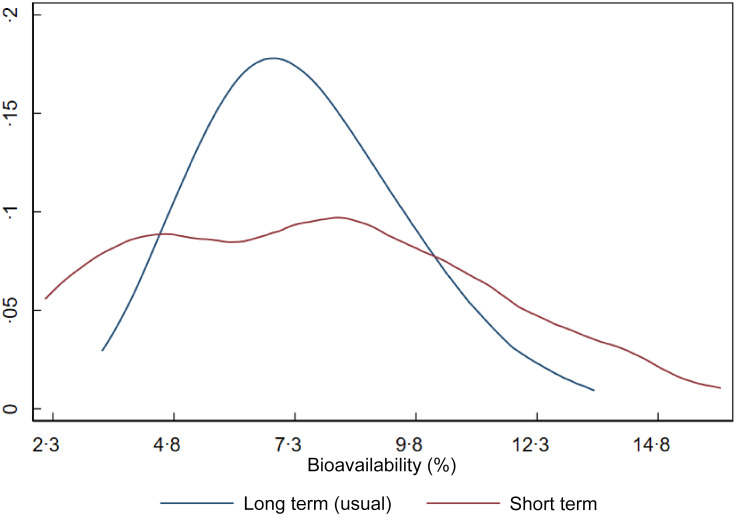
Short-term and long-term iron bioavailability distributions in Brazilian children aged 7–23 months.

Figure 2 shows the distribution of iron bioavailability in the sample. The red curve represents bioavailability based on a single day of dietary intake (1-d 24-h recall), while the blue curve represents usual (long-term) bioavailability after adjustment for within-person day-to-day variation. Although the means are similar, the shape and dispersion of the curves differ markedly, impacting the CV of dietary iron requirements. Since dietary requirements are intended to reflect long-term intake rather than single-day values, we used the parameters (mean and variance) from the usual bioavailability distribution in estimating the NRV.

Finally, Table 4 presents the NRV estimated for Brazilian children. The AR for children aged 7–11 months and 12–23 months were 11 mg/d (CV: 30 %) and 8 mg/d (CV: 30 %), respectively. The corresponding PRI were 20 mg/d for 7–11 months and 14 mg/d for 12–23 months. Table 4 also compares these estimates with those from the IOM and EFSA.

**Table 4.** Iron requirement, average requirement and population recommended intake for children 7–23 months old for Brazil, Europe and the USA/Canada

	7–11 months			12–23 months		
	Brazil	EFSA	IOM	Brazil	EFSA	IOM
Iron bioavailability (%)	7·5	10	10	7·5	10	18
EAR/AR (mg/d)	11	8	6·9	8	5	3
RDA/PRI (mg/d)	20	11	11	14	7	7

EFSA, European Food Safety Authority; IOM, Institute of Medicine; EAR, estimated average requirement; AR, average requirement; PRI, population reference intake.

## Discussion

In this study, we adapted the iron reference intake values for Brazilian children aged 7–23 months. The NRV we estimated were higher than those published by the IOM^(20)^, EFSA^(21)^ and other countries that have established their own reference values, such as India^(8)^ and Venezuela^(6)^.

Ideally, the establishment or adaptation of NRV in a population should be informed by locally specific metabolic, anthropometric and dietary data. However, such information is often limited to a small number of studies, mostly conducted in American or European populations. This was the case for parameters such as basal iron losses and iron requirements for growth, for which we adopted the same values used by EFSA^(21)^. This reliance on external data represents a potential limitation when deriving country-specific NRV. Furthermore, we found no evidence on population-specific variations in basal iron losses; thus, the applicability of these parameters to Brazilian children remains uncertain. Nevertheless, as basal losses are not expected to vary substantially across populations, we consider that the uncertainty involved in using these values for Brazilian children is likely similar to that when applying them to other populations, such as in the USA or Europe. These remain the best available estimates for population-level NRV. We identified a few other studies that adapted NRV, such as those conducted in India^(8)^ and Venezuela^(6)^, which also used the same physiological parameter assumed by the IOM.

The IOM and EFSA committees adopted different growth standards to estimate their respective AR. The IOM relied on growth curves proposed by Frisancho (1990)^(44)^, which were based on data from US children. EFSA, on the other hand, used WHO growth standards (2006)^(17)^, which were developed from a multicentre study including data from Brazil, Ghana, India, Norway, Oman and the USA. It is well established that all children under 5 years of age have the same potential to grow if provided with a healthy environment, adequate care and essential nutrients^(45)^. This supports the use of a single reference weight distribution across countries. Another important decision in our study was to use a reference growth standard rather than the actual weight distribution of the Brazilian population. This is justified by the conceptual nature of NRV, which are intended to represent the needs of a healthy population. Children with body weights significantly below or above the reference may be considered to have suboptimal health status. Moreover, if a country’s average weight-for-age index is lower than the reference, the estimated iron requirements would reflect a population with potentially compromised health. Few studies have established NRV in low- and middle-income countries.

Iron bioavailability was the most influential factor explaining the differences between our NRV and those of other authorities. In our study, iron bioavailability in Brazilian children was estimated at 7·5 %, considerably lower than the values assumed by US guidelines (10 % and 18 % for children aged 7–11 and 12–23 months, respectively) and EFSA (10 %). The lower bioavailability in Brazil can be attributed to a higher reliance on plant-based foods compared to diets in the USA and Europe. Cereals and beans, which are staples in Brazil, are rich in non-heme iron, a form of iron with low absorption rates (approximately 5 % in our study). In contrast, heme iron, which is absorbed at a rate of about 25 %, is found in animal-source foods that are consumed less frequently in Brazil. Additionally, unlike in the USA and Europe, iron-fortified breakfast cereals are not widely consumed by Brazilian children^(46)^. Our analysis also incorporated the variability in iron bioavailability when calculating NRV. We estimated the CV for the AR and PRI, rather than applying a fixed bioavailability assumption as done by EFSA and IOM. This approach introduced greater variance in the dietary requirement estimates, resulting in a CV of 30 %, compared with 20 % (EFSA) and 10 % (IOM).

This study has some limitations. First, there is a lack of local data for estimating physiological parameters such as iron losses and the iron required for growth. While this is a common challenge in establishing NRV for most countries, we found no evidence that such parameters vary substantially across populations. Second, we estimated iron bioavailability using an algorithm developed for adults. This limitation is also shared by other NRV for children, and to our knowledge, the model we used remains the most appropriate and up-to-date reference on the subject. Third, our bioavailability estimates were not derived from a nationally representative sample. However, the data came from a representative sample of children in the city of Rio de Janeiro, which is one of the largest cities in Brazil. This is a very cosmopolitan city comprising people of different races, colours, social strata and with heterogeneous dietary patterns and consequently nutrient intake.

Despite these limitations, this study represents the first effort to address a gap in nutrient intake recommendations in Brazil, both from an academic and clinical practice standpoint. For decades, nutritionists and researchers in Brazil have relied on IOM NRV without adequately evaluating their applicability to the local context. This study is also among the few initiatives to adapt national NRV using the harmonisation approach, which enhances methodological transparency and supports reproducibility and comparability across populations.

In conclusion, the AR for children aged 7–11 months and 12–23 months were 11 mg/d and 8 mg/d, respectively. The corresponding PRI were 20 mg/d and 14 mg/d, both higher than the values recommended by the IOM and EFSA. Iron bioavailability was the key factor accounting for these differences. The application of the harmonisation approach proved to be a valuable method for adapting NRV to national contexts.
